# Modified Aortic Valve Reimplantation in Patients with Acute Type A Aortic Dissection

**DOI:** 10.21470/1678-9741-2024-0056

**Published:** 2025-10-27

**Authors:** Sergey Yurevich Boldyrev

**Affiliations:** 1 Kuban State Medical University of the Ministry of Health of the Russian Federation, Krasnodar, Krasnodar, Russian Federation

**Keywords:** Aortic Valve, Aortic Valve Insufficiency, Aorta, Thoracic, Follow-Up Studies, Hospital Mortality, Cardiopulmonary Bypass, Constriction, Aortic Dissection, Replantation.

## Abstract

**Introduction:**

Choosing a surgical technique in patients with acute type A aortic dissection is still a debatable issue. In patients with massive aortic root destruction, the Bentall procedure is a gold standard. Aortic valve reimplantation is a reliable alternative, especially in patients with the preserved anatomy of aortic valve leaflets.

**Objective:**

To compare the results of modified valve sparing procedure and composite root replacement in patients with acute type A aortic dissection.

**Methods:**

In total, 62 patients were included in this study. Of those, 27 patients underwent aortic valve reimplantation, and 35 had the Bentall procedure with the Kouchoukos modification.

**Results:**

Preoperative demographics and clinical characteristics were analyzed in both groups. Similar indicators of preoperative malperfusion were observed in both. Cardiopulmonary bypass time (P = 0.125) and aortic clamping time (P = 0.001) were longer (≈ 30 minutes) in the reimplantation group while the time of circulatory arrest was longer in the Bentall group (P = 0.290). Hospital mortality rates were 8.3% in the reimplantation group and 22.9% in the Bentall group. During the long-term follow-up period, there were six (25%) deaths in the reimplantation group and 10 (28.6%) deaths in the Bentall group. The aortic regurgitation degree was stable in all cases up to the moment of last contact with the patients.

**Conclusion:**

Modified aortic valve reimplantation shows good immediate and long-term outcomes in patients with acute type A aortic dissection.

## INTRODUCTION

**Table:** 

Abbreviations, Acronyms & Symbols	
BSA	= Body surface area
CABG	= Coronary artery bypass grafting
LAD	= Left anterior descending artery
LVEF	= Left ventricular ejection fraction
RCA	= Right coronary artery

Acute type A aortic dissection of the ascending aorta accounts for around 80 - 90% of all acute aortic syndromes^[1]^. According to data from numerous sources, the ascending aorta dissection is identified in approximately 2.5 - 5 per 100,000 people per year^[2^,^3]^. The risk of death if untreated remains high. Open surgical reconstruction of the ascending aorta is a life-saving operation and remains a standard treatment for patients with acute type A aortic dissection^[4]^. The choice of a surgical technique for patients with acute type A aortic dissection is an open question, and we should therefore admit the importance of studying the native aortic valve reimplantation technique as well as the quality of life of patients in the immediate and long-term postoperative periods.

## METHODS

This study was approved by the Ethics Committee of the SBIPH Scientific Research Institute - Ochapovsky Regional Clinical Hospital №1 State Budgetary Healthcare Institution of the Ministry for Health Care of Krasnodar Region (#164), and it was carried out in accordance with the requirements of medical insurance.

### Patients

All the data of patients with acute aortic dissection who were admitted to the Research Institute - Ochapovsky Regional Clinical Hospital from 2003 to 2023 have been analyzed. Patients with acute type A aortic dissection who underwent emergency surgery were selected for the analysis. In total, 62 patients were included in the study, identified, and stratified by the risk of the initial severity of their condition. Of those, 27 patients underwent modified aortic valve reimplantation and 35 had the Bentall operation. The criteria for excluding valve reimplantation were the pathology of the aortic valve leaflets, presence of fenestrations, and extremely severe haemodynamic condition of the patient.

### Surgical Technique

All procedures were performed through a median sternotomy using cardiopulmonary bypass. A plan for cannulation was made based on the anatomy of the dissection and at the discretion of the operating surgeon based on the volume of the proposed aortic reconstruction. Preference for arterial cannulation was given mainly to the brachiocephalic trunk or the ascending aorta under ultrasound monitoring. Crystalloid cardioplegia was used in all cases, and antegrade cerebral perfusion was performed during circulatory arrest to protect the brain. All patients underwent near-infrared spectroscopy, and measurements were made using an INVOS™ system (Somanetics). The level of hypothermia was selected individually for each patient and ranged from 20 to 26°C. Depending on a surgeon's preferences, three versions of the reimplantation technique were applied: Seattle, David V/Miller, and the proprietary modification. The reimplantation technique started with the Seattle modification, which was mainstream at that time, and only two patients were operated on in this way. Fitting of the vascular graft was performed by adding 3 mm to a measured fibrous annulus, and the base of the vascular graft was trimmed in such a way as to be positioned opposite to the commissures, creating new sinuses^[5]^.

The David V/Miller technique was used in five cases. Multiple^[6^-^8]^ U-shaped sutures were placed on the fibrous annulus. When selecting the size of the graft, 6 - 8 mm were added to the diameter of the fibrous annulus, leaving more space for the aortic valve leaflets. The vascular graft was narrowed in the proximal part with a polyester filament on the selected fibrous annulus measuring tool. In addition, vascular graft plication was carried out on three sides with separate sutures at the level of the sinotubular junction in order to shape neosinuses^[9]^.

In this proprietary modification, the planned intervention on the aortic arch was performed first, then cardiopulmonary bypass was recommenced, and the repair at the root was started.

The coronary arteries were mobilized from all abnormal or dissected tissue, leaving a border of 4 - 5 mm. During this procedure, the tissue structure and the presence of ruptures were evaluated, and if dissection extended to the arterial ostia, the wall layers were juxtaposed with the use of additional felt pads or plication was carried out.

Measurement of the fibrous annulus is carried out by inserting the original device into the left ventricle through the aortic ring, the size of which corresponds to one of the dimensions of the working part of the device^[10]^. The vascular graft was selected as in the David V/Miller method according to the following formula: size of the vascular graft = size of the selected device + 8 mm. However, our method of narrowing the proximal part of the vascular graft involves suturing it with two filaments horizontally, retracting from the edge of the prosthesis 1 - 2 mm, in the form of a purse-string suture so that the sutures are placed at a distance of 3 mm from each other in a checkered order. This results in a margin with a width up to 5 mm being obtained at the proximal end of the vascular prosthesis. Then three U-shaped sutures only were placed transmurally moving from inside to outside using pads located below the base of the leaflets. After stitching the fibrous annulus, the aortic valve was reimplanted and fixed into the vascular prosthesis. The coronary buttons were implanted into the graft in turn.

Patients who did not match the inclusion criteria had a traditional root replacement using a mechanical valve conduit according to the Bentall method with the Kouchoukos modification^[11]^. In most cases, at the end of the operation, biological glue was used for additional sealing of the anastomoses.

### Immediate and Long-Term Results

Mortality from any cause and the degree of increasing regurgitation were issues of major interest. All patients with preserved native valves underwent routine annual examinations with echocardiography to assess the evolution of aortic regurgitation.

### Statistical Analysis

Data analysis was performed using the IBM Corp. Released 2019, IBM SPSS Statistics for Windows, version 26.0, Armonk, NY: IBM Corp. statistical software.

The statistical description of continuous quantitative variables is presented as the mean ± standard deviation, categorical variables are described as an absolute and relative frequency (absolute n [% from n]). The clinical signs of the David and Bentall groups were compared: in the event of normally distributed continuous signs using the Student’s t-test for independent samples, and in the event of continuous signs that do not match normal probability laws using the Mann-Whitney U test. Review of the data distribution for compliance with the normal laws was carried out using the Kolmogorov-Smirnov test. Comparison of the frequency of categorical (binary) features was performed using z-test for further comparison of proportions.

The *P* < 0.05 value indicated statistically significant differences in the groups being compared. The Kaplan-Meier method was applied to assess freedom from regurgitation over a 15-year period following surgery.

## RESULTS

Sixty-two patients were included in this study, of which 27 had aortic valve reimplantation according to one of the modifications. Table 1 shows the demographics and clinical characteristics of the patients. In both groups, there were more male patients than female. The average age was 49 years. The body surface area of the patients in both groups demonstrated no significant difference (*Р*=0.949). In addition, there was no significant difference regarding the presence of concomitant diseases in the groups. This study included patients with Marfan syndrome, with three in the reimplantation group and nine in the Bentall group. There were also patients with a bicuspid aortic valve, one in the reimplantation group and six in the Bentall group. The ejection fraction of the left ventricle was preserved in both groups in most cases. In the Bentall group, the average diameter of the ascending aorta was larger than in the reimplantation group (*Р* = 0.036). Aneurysms of the aortic arch were observed in four cases, one in the reimplantation group and three in the composite grafting group. In total, there were 42 patients with De Bakey type I aortic dissection, and in 20 cases there was De Bakey type II. No significant difference in the clinical and radiological manifestation of malperfusion was observed.

**Table 1:** Preoperative variables according to group.

		Total	David	Bentall	*P*-value
		N = 62 (%)	N = 27 (%)	N = 35 (%)	
Sex	Male	48 (77.4)	22 (81.5)	26 (74.3)	0.555^а^
	Female	14 (22.6)	5(18.5)	9 (25.7)	
Age (years)		48.98 ± 11.36	50.11 ± 10.2	48.11 ± 12.28	0.497^b^
BSA (m^2^)		1.97 ± 0.24	1.98 ± 0.24	1.97 ± 0.24	0.949^b^
Concomitant	Hypertension	30 (48.4)	10 (37.0)	20 (57.1)	0.119
	Diabetes	2 (3.2)	2 (7.4)	0	0.105
	Hypertension & diabetes	6 (9.7)	1 (3.7)	5 (14.3)	0.165
Marfan syndrome		12 (19.4)	3 (11.1)	9 (25.7)	0.152
Bicuspid aortic valve		7 (11.3)	1 (3.7)	6 (17.1)	0.101
Aortic annulus (mm)		25.32 ± 2.33	25.00 ± 2.35	25.57 ± 2.32	0.343^b^
LVEF (%)		51.74 ± 5.35	51.89 ± 5.32	51.63 ± 5.4	0.914^c^
Ascending aorta diameter (mm)		60.61 ± 12.39	57.41 ± 11.61	63.09 ± 12.57	0.036^с^
Aortic arch aneurysm		4 (6.5)	1 (3.7)	3 (8.6)	0.440
De Bakey type	I	42 (67.7)	18 (66.7)	24 (68.6)	1.0^а^
	II	20 (32.3)	9 (33.3)	11 (31.4)	
Symptomatic malperfusion	Cerebral	5 (8.1)	3 (11.1)	2 (5.7)	0.442
	Renal	2 (3.2)	1 (3.7)	1 (2.9)	0.861
	Coronary	3 (4.8)	1 (3.7)	2 (5.7)	0.718
	Lower extremities	2 (3.2)	1 (3.7)	1 (2.9)	0.861
	Renal & lower extremities	1 (1.6)	1 (3.7)	0	0.255
X-ray malperfusion	Cerebral	4 (6.5)	3 (11.1)	1 (2.9)	0.197
	Renal	3 (4.8)	2 (7.4)	1 (2.9)	0.418
	Coronary	4 (6.5)	0	4 (11.4)	0.072
	Lower extremities	2 (3.2)	2 (7.4)	0	0.105
	Cerebral & renal	4 (6.5)	1 (3.7)	3 (8.6)	0.440
	Cerebral & lower extremities	1 (1.6)	0	1 (2.9)	0.376
	Coronary & renal	1 (1.6)	1 (3.7)	0	0.255
	Cerebral, renal & lower extremities	6 (9.7)	4 (14.8)	2 (5.7)	0.233

a the exact Fisher criterion was used;

b the Student's *t*-test was used for two independent samples;

c the Mann-Whitney U test was used; in other cases, the *z*-test was used to compare proportions

BSA=body surface area; LVEF=left ventricular ejection fraction

The period of cardiopulmonary bypass and aortic clamping was longer in the reimplantation group, while the time of circulatory arrest was longer in the Bentall group (Table 2). The mean time of clamping of the aorta was about 30 minutes longer in the reimplantation group. All patients in the Bentall group underwent replacement of a bileaflet mechanical valve conduit. In the reimplantation group, coronary artery bypass grafting was performed in three cases due to extended dissection of the coronary ostia. The frequency of complete aortic arch replacement was higher in the Bentall group. In the early postoperative period in the Bentall group, decreased cardiac output requiring increased time on cardiac support, respiratory failures requiring prolongation of mechanical ventilation, cerebrovascular disorders, and resternotomy for further debridement on the first postoperative day were more common (Table 3). The hospital mortality rate was lower in the reimplantation group, with only two deaths compared to eight in the Bentall group. In one case in the reimplantation group, diffuse tissue bleeding was observed as a result of the development of consumption coagulopathy and subsequent myocardial infarction, and a second patient required inotropic support in the postoperative period due to severe heart failure that subsequently led to their death.

**Table 2:** Operative variables according to group.

		Total	David	Bentall	*P*-value
		N = 62 (%)	N = 27 (%)	N = 35 (%)	
Cardiopulmonary bypass time (min.)		233.68 ± 87.51	247.30 ± 72.26	223.17 ± 97.4	0.125^b^
Aortic clamping time (min.)		144.37 ± 43.90	164.63 ± 32.30	130.69 ± 42.75	0.001^а^
Arrest time (min.)		30.89 ± 25.21	25.59 ± 19.97	34.97 ± 28.2	0.290^b^
Hypothermia (°C)		24.29 ± 3.11	24.59 ± 3.1	24.06 ± 3.1	0.506^a^
Arterial cannulation	Ascending aorta	32 (51.6)	12 (44.4)	20 (57.1)	0.325
	Brachiocephalic trunk	14 (22.6)	8 (29.6)	6 (17.1)	0.247
	Axillary artery	4 (6.5)	3 (11.1)	1 (2.9)	0.197
	Femoral artery	12 (19.4)	4 (14.8)	8 (22.9)	0.427
Vein cannulation	Right atrium	59 (95.2)	27 (100)	32 (91.4)	0.121
	Femoral vein	3 (4.8)	0	3 (8.6)	0.121
Total arch replacement		4 (6.5)	1 (3.7)	3 (8.6)	0.440
Hemiarch replacement		45 (72.6)	20 (74.1)	25 (71.4)	0.815
Concomitant CABG	LAD	1 (1.6)	1 (3.7)	-	-
	RCA	2 (3.2)	2 (7.4)	-	-
Modification (David)	David V/Miller	-	5 (18.5)	-	-
	Kuban Cuff^*^	-	20 (74.1)	-	-
	Seattle	-	2 (7.4)	-	-
Vascular graft diameter (mm)		-	30.22 ± 2.24	-	-
Conduit size (Bentall) (mm)		-	-	25.86 ± 1.96/28.8 ± 1.9	-

a the Student's *t*-test was used for two independent samples;

b the Mann-Whitney U test was used; in other cases, the *z*-test was used to compare proportions;

* Kuban Cuff modified reimplantation

CABG=coronary artery bypass grafting; LAD=left anterior descending artery; RCA=right coronary artery

**Table 3:** Postoperative outcomes according to group.

	Total	David	Bentall	*P*-value
	N = 62 (%)	N = 27 (%)	N = 35 (%)	
Low cardiac output	11 (17.7)	3 (11.1)	8 (22.9)	0.232^a^
Respiratory failure	10 (16.1)	4 (14.8)	6 (17.1)	0.809^a^
Cerebrovascular accident	3 (4.8)	1 (3.7)	2 (5.7)	0.718^a^
Reoperation for bleeding	7 (11.3)	2 (7.4)	5 (14.3)	0.399^a^
In-hospital mortality	10 (16.1)	2 (7.4)	8 (22.9)	0.103a
Total mortality	16 (25.8)	6 (22.2)	10 (28.6)	0.571^a^

a the *z*-test was used to compare proportions

**Fig. 1:**
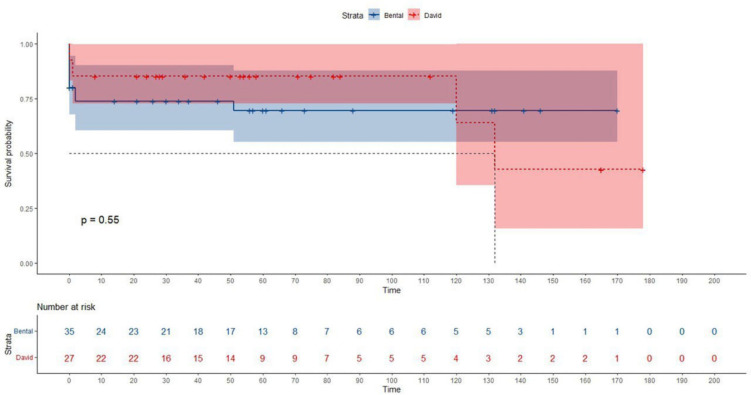
Analysis of the risk of death in the Bentall (blue) and David (red) group.

**Fig. 2:**
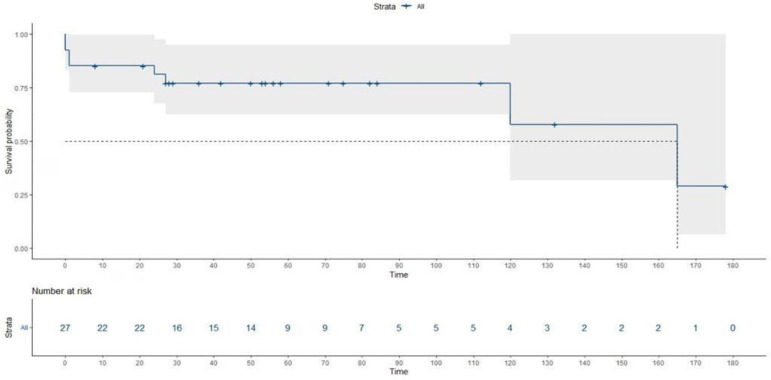
Degrees of aortic valve insufficiency in the postoperative period.

Further follow-up of the patients showed that in the reimplantation group, two more deaths occurred in the 90-day postoperative period. One patient died 78 days after the procedure with pulmonary artery embolism being the cause of death. The second patient developed acute heart failure 62 days after the operation. The mortality rate from all causes was lower in the reimplantation group compared to the Bentall group (*P* = 0.332) (Figure 1). Figure 2 shows the outcomes of the postoperative follow-up of patients with echocardiography to assess the degree of increased aortic regurgitation. The degree of valve insufficiency did not increase in any of the cases up to the time of the last examination of the patient.

## DISCUSSION

According to the American College of Cardiology/American Heart Association guidelines for the diagnosis and treatment of aortic diseases and the American Association for Thoracic Surgery consensus on the surgical management of acute type A aortic dissection, in most cases (evidence level IВ) it is recommended to use valve-sparing operations, except in cases with a dissection extending below the sinotubular junction^[4^,^12]^. Supracoronary aortic grafting is a recommended technique because of the reduced time of myocardial ischemia, cardiopulmonary bypass, and minimum dissection of altered tissues in patients in an acute stage, as it reduces the risk of hemorrhagic complications. However, this technique means the risk of native root expansion in the future, which could require a further intervention^[13^,^14]^.

The Bentall procedure has proven its effectiveness for several decades, and it remains the gold standard for acute type A aortic dissection because of its safety and reproducibility (evidence level IB), although the use of a mechanical valve condemns patients to lifelong anticoagulant therapy, potential haemorrhagic complications, and an increased risk of thromboembolism^[4^,^6]^.

Considering that in most patients with acute dissection the aortic valve leaflets remain intact, some techniques have been offered for preserving the native valve^[7^,^8]^. In the Yakub remodeling technique, the lack of stabilization of the valve ring could lead to recurrent aortic insufficiency, and a relatively long line of anastomosis increases the risk of hemorrhagic complications, which is why the number of operations performed using this technique in cases of acute aortic dissection is ultimately insignificant^[15]^. This fact limits the experience of using this operation, especially in cases of tissue dissection.

A revolutionary reimplantation technique originally described by David et al. in 1992 has advantages, as there is no need for long-term anticoagulant therapy following complete restoration of the aortic root, which makes it a recommended treatment, especially in young patients. If this technique is used in a comprehensive aortic center, we can expect excellent results without repeated procedures, with follow-up from five to 15 years^[16]^.

However, the use of aortic valve reimplantation procedures in cases of acute aortic dissection is currently limited^[17^,^18]^. The International Registry of Acute Aortic Dissection reported 682 patients who underwent surgery in 18 centres, and aortic valve reimplantation was performed in only 5.8% of patients^[19]^. The German Registry of Acute Aortic Dissection Type A presented data from 56 centres, where 8.2% were assigned to aortic valve reimplantation techniques^[20]^. The reasoning for this is that the procedure of valve reimplantation is technically complicated and is therefore associated with longer operating times and requires extensive dissection of altered tissues, which in the acute period of this pathology can be a difficult task even for experienced surgeons^[21]^. The combination of these factors dictates a certain tactical approach, in particular concerning the proper selection of patients for the reimplantation procedure in the conditions of acute aortic dissection.

In this paper, the experience of surgical treatment of acute aortic dissection in a single facility has been presented. We have been performing valve implantations using the David method with our proprietary modification as the preferred intervention over a period of several years^[22^,^23]^. Using our technique, the number of sutures needed to fix the graft is reduced to three, which expedites this stage of the intervention. In addition, for better stabilization of the fibrous annulus, two lines of sutures are placed when modelling the proximal part of the vascular prosthesis. It should be mentioned that in cases where modified reimplantation was carried out, there were no cases of an increased degree of aortic regurgitation during the follow-up period, which lasted for up to 15 years.

### Limitations

This study has limitations. First of all, it was retrospective analysis; second, the low number of the studied cases and low volume of events for the main valve-related outcomes.

The small number of events associated with endpoints of death and repeated aortic valve surgery did not allow for any reliable statistical analysis of the factors associated with these events.

## CONCLUSION

The data in this study confirm the feasibility of surgical treatment of acute aortic dissection with preserved aortic valve leaflet anatomy using the modified reimplantation method in selected patients. Our results show good long-term survival rates, as well as a complete absence of increases in the level of aortic regurgitation. It should be emphasized that these types of operations for acute aortic dissection must be performed in comprehensive aortic centers by highly skilled surgeons.

## Data Availability

The author declares that he does not have a special link for data repository but is ready to share it.

## Preprint

The authors declare that a preprint version of this article is available on medRxiv at https://www.medrxiv.org/content/10.1101/2024.02.07.24302455v1.

## Artificial Intelligence Usage

The author declares that no artificial intelligence tool was used in the preparation of this article.
